# The complete chloroplast genome of *Hibiscus Taiwanensis* (Malvaceae)

**DOI:** 10.1080/23802359.2019.1640084

**Published:** 2019-07-13

**Authors:** Xin-Rui Xu, Song-Dong Zhou, Xiao-Qing Shi

**Affiliations:** a Key Laboratory of Bio-Resources and Eco-Environment of Ministry of Education, College of Life Sciences, Sichuan University, Chengdu, P. R. China;; b Chengdu Institute of Landscape Architecture, Chengdu, Sichuan, P. R. China

**Keywords:** *Hibiscus taiwanensis*, chloroplast, genome

## Abstract

*Hibiscus taiwanensis* S. Y. Hu is an ornamental plant of *Hibiscus*, native to Taiwan. Here, we reported the complete chloroplast genome of *H. taiwanensis*. The chloroplast genome of *H. taiwanensis* was 161,056 bp in length, containing a couple of inverted repeat (IR) regions of 26,300 bp, a large single-copy (LSC) region of 89,538 bp and a small single-copy (SSC) region of 18,918 bp. The complete chloroplast genome annotation revealed a total of 131 genes, including 85 protein-coding genes, 7 rRNA genes, and 37 tRNA genes. The entire GC content was 36.9%. Phylogenetic tree analyses indicated that *H. taiwanensis* was closely clustered with *H. rosa-sinensis* and *H. syriacus*.

*Hibiscus taiwanensis* S. Y. Hu, a deciduous tree or shrub, which belongs to the genus *Hibiscus* of the family Malvaceae. This plant, 3–8 m high, is not stellate, but densely strigose and scabrous (Tang et al. 2007). The *H. taiwanensis* grows everywhere below the altitude of 1200 m all over Taiwan and has been distributed elsewhere in Southeast Asia (Lim 2014). It is introduced as an ornamental plant by the world's major botanical gardens. Its blossom is not only seductive but also esculent. The stem and root of *H. taiwanensis* have been used as antifungal, analgesic, antipyretic, anti-inflammatory, and anthelmintic agents in traditional Chinese medicine (Gan 1965). Previous studies on *H. taiwanensis* have focused on its chemical composition and medicinal value (Lim 2014). However, there are a few reports on the taxonomy and phylogeny of *H. taiwanensis*. Here, we first report the complete chloroplast genome sequence of *H. taiwanensis* to provide genomic sources for further researching phylogenetic relationships of *Hibiscus*.

The fresh and healthy leaves were collected from the Chengdu Botanical Garden (30°45′52″N, 104°8′11″E), Sichuan Province, China. Voucher specimens were deposited in the Herbarium of Sichuan University (SZ, XXR20181221). The total genomic DNA was extracted from the above leaves with the modified CTAB method (Doyle and Doyle 1987). The total genomic DNA was sequenced using the Illumina Hiseq Platform (Illumina, San Diego, CA, USA). We used the raw data to assemble the complete chloroplast genome by NOVOPlasty (Dierckxsens et al. 2017), with the complete chloroplast genome of *H. rosa-sinensis* as the reference (GenBank accession no. MK382984). We annotated the assembled chloroplast genome via Geneious 11.0.4 with the sequence of *H. rosa-sinensis* as the reference and corrected the annotation result manually (Kearse et al. 2012). Finally, the complete chloroplast sequences of 13 species which belong to Malvales were aligned by MUFFT (Katoh et al. 2002). The sequences which have been aligned were applied to build the maximum-likelihood (ML) tree with 1000 bootstrap replicates by RaxML (Stamatakis 2006).

The circular chloroplast genome of *H. taiwanensis* was 161,056 bp in length (GenBank accession no.MK937807), divided into four regions. A couple of inverted repeat (IR) regions of 26,300 bp separated by the large single-copy (LSC) region of 89,538 bp and small single-copy (SSC) region of 18,918 bp. The chloroplast genome detected a total of 131 genes, including 85 protein-coding genes, 7 rRNA genes, and 37 tRNA genes. The entire GC content of *H. taiwanensis* cp genome was 36.9% with the corresponding values of LSC, SSC, and IR regions being 34.7%, 31.5%, and 42.6%, respectively.

As a few complete chloroplast sequences of Malvaceae have been released, we choose the complete chloroplast sequences of 13 species which belong to Malvales to construct the phylogenetic tree. The result showed that *H. taiwanensis* is closely clustered with *H. rosa-sinensis* and *H. syriacus* (Figure 1), which was in accordance with early studies (Tang et al. 2014). This complete chloroplast genome can be further used for studying the value of *H. taiwanensis* and the phylogenetic relationships among the genus *Hibiscus* L.

**Figure 1.**
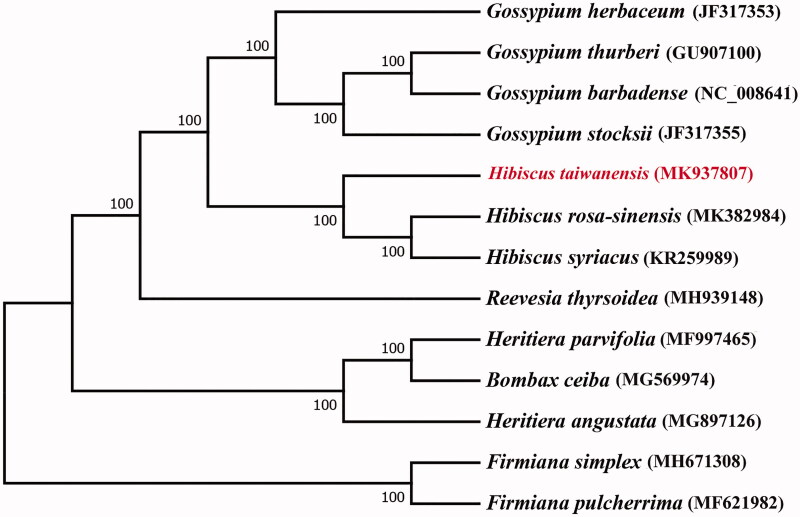
ML phylogenetic tree of *H. taiwanensis* with 12 species of Malvales was constructed by chloroplast genome sequences. Numbers on the nodes are bootstrap values from 1000 replicates. Two species of *Firmiana* were selected as outgroup.
